# 

**Published:** 1897-08

**Authors:** 


AUGUST, 1897.

### THE AMERICAN JOURNAL OF DENTAL SCIENCE.

EDITED BY

F. J. S. GORGAS, M. D., D. D. S.
RICHARD GRADY, M. D., D. D. S.

Vol. 31. THIRD SERIES No. 4.

BALTIMORE:
SNOWDEN & COWMAN MFG. CO., 9 W. Fayette St.

LONDON:

TRUBNER & CO., 60 Paternoster Row.

Entered at the Post Office at Baltimore, Md., as Second-class Matter.

PUBLISHED MONTHLY.

$2.50 PER ANNUM.

PAYABLE IN ADVANCE.

